# Society Meetings

**Published:** 1911-12

**Authors:** 


### SOCIETY MEETINGS

### Important Amendments

The Medical Society of the County of Erie held an adjourned meeting October 20, 1911, at which Dr. Edward E. Haley, Chairman of the Committee on Contract Work, submitted a detailed report of his committee, as a result of which the following resolution and amendments to the by-laws, submitted by the committee, were adopted:

1. *Resolved,* That the proposed amendment shall not apply to present contracts, but shall prohibit any extension or renewal of the same.

2. Add a section to Chapter II of the Constitution and By-laws to be known as Section 13, and to read as follows:

"No one shall become a member of this Society or continue as such, who engages in contract-work, unless it be governmental in character, but this shall not prohibit an agreement for a particular case nor apply to examinations for an adequate fee."

Also the following recommendations:

1st. That the President appoint, from time to time, at his own discretion, special committees to use their influence on any man who continues this inethical practice, whether he be a member of this Society or not.

2d. That a special committee be appointed to assist the membership committee to devise ways and means to induce all desirable men to join the Society.

3d. That the proper authorities of this Society bring this matter to the attention of the State Society for the purpose of gaining its support.

The Secretary was directed to send a copy of these resolutions and amendments to each member of this Society, and to also send a copy to every lodge, organization, firm or corporation employing medical service by contract.

Amendments to the By-laws creating a sinking fund were adopted as follows:

> Amend Chapter 12 so that caption shall read "Dues and Finances" instead of "Dues."
>
> Amend by adding to Chapter 12 two sections to be known as Sections 4 and 5 which shall read as follows:
>
> Sec. 4. After paying all outstanding floating indebtedness, all money derived from dues and in excess of one hundred dollars ($100.00) that remains in the treasury at the close of each year (December 31), as well as such other moneys as this Society may, from time to time, direct, shall be deposited in savings institutions designated by the Council, to the credit of a fund to be known as the "Permanent Fund of the Medical Society of the County of Erie."
>
> Sec. 5. No money shall be withdrawn from the Permanent Fund of the Medical Society of the County of Erie, except by a two-thirds vote of the members present at a regular meeting, and provided that notice of intent to move for such withdrawal shall have been approved by the Council and a copy thereof sent to each member with the notice for the meeting at which such withdrawal will be considered."
>
> Nominations of officers were made, the election to take place at the annual meeting on the third Monday in December.

---

### Phi Alpha Gamma Convention

SIXTEEN years ago, seven students in the New York Homeopathic Medical College and Flower Hospital got together to form a small social club for the mutual benefit of its members. By one of those peculiar coincidences that we often observe, similar small clubs were formed in Boston University Medical School and in Hahnemann Medical College of Philadelphia, neither one of which knew of the organization in New York.

In 1897 these three local clubs were fused into one intercollegiate fraternity under the name of the first one to be formed.

The fraternity idea spread rapidly, until within a very few years chapters of the new society were founded in over a dozen colleges scattered from the Atlantic to the Pacific, and its members now number, including undergraduates and alumni, over two thousand.

Within the last two or three years there have been formed Alumni Chapters of the organizations in cities where sufficient members were located. Such chapters now exist in Boston, New York, Philadelphia, Chicago, Buffalo, Rochester and Cleveland. The convention of the fraternity just ended is the first one to meet as the guests of an Alumni Chapter, all previous ones having been held in some city where there existed an active chapter in a college.

The Grand Chapter meets annually in convention, each active chapter sending two delegates and each alumni chapter sending one. The officers of the sixteenth convention are: Grand President, Dr. A. D. Carpenter, Buffalo; Grand Vice-President, Dr. H. B. Kinyon, Ann Arbor, Mich.; Grand Secretary-Treasurer, Dr. Richard H. Street, Chicago. The officers of the Buffalo Alumni Chapter are Dr. G. R. Critchlow, President; Dr. C. L. Hyde, Secretary Treasurer. The local chapter numbers fifteen members, several of them residents of surrounding towns.

The business of the convention occupied two days and a half, November 16, 17 and 18. On Thursday evening was held a smoker and vaudeville performance at the German-American Café, and the annual Banquet was given on Friday evening at Hotel Statler. The next convention will be held at Cleveland as the guests of the Cleveland Alumni Chapter.

G. R. CRITCHLOW.

### Annual Meeting Sanitary Officers of the Association of New York State

THE New York State Sanitary Officers' Association, made up of the fourteen hundred Health Officers of the State, held its 3d annual meeting at the Hotel Astor, New York City at 2.00 o'clock Tuesday afternoon, October 24. The officers for the ensuing year were elected as follows:

Frank Overton, M.D., Patchogue, President.

Vice-Presidents:

Louis M. Brown, M.D., Purdy's Station.
Daniel S. Burr, M.D., Binghamton.
Louis E. Couch, M.D., Nyack.
O. J. Hallenbeck, M.D., Canandaigua.
William B. Stanton, M.D., Webster, Treasurer.
Montgomery E. Leary, M.D., Rochester, Secretary.

Over one hundred members were present and an exceedingly interesting program was presented. Several of the speakers dwelt upon their experiences in the practical application of the present sanitary laws and it was the opinion of the Association that the present code is inefficient and inadequate. Health Officers are afforded practically no real protection. One instance was cited where a Health Officer was put to the expense of nearly $1,000.00 in defending a suit which the court eventually threw out—"No cause for action."

The Attorney General has expressed an opinion that the local Boards of Health—Village or Town—have no right under the present law to incur an expense for articles destroyed by Health Officers. The position of a Health Officer is thus made not only possibly expensive but very incapable of accomplishing much where opposition is not.

*Revision of the Sanitary Code.*—The State Department of Health through Commissioner Eugene H. Porter, Deputy Commissioner William H. Howe and Secretary Alec H. Seymour, has pledged its hearty support in co-operating towards securing the necessary revision. President Frank Overton is selecting a Legislative Committee of five with himself and the secretary as ex-officio members, which will immediately take up this rather formidable task. It will require not only much labor but much time to complete. The codes of various states will be studied and the good points of each adopted. This will be the beginning of placing New York in the front rank in sanitary work.

This is the first instance we know of where the actual revision of the sanitary laws will be undertaken by the organized effort of the Health Officers in co-operation with the State Department of Health. It is hoped by adopting this means to secure a nearer approach to the ideal than has been accomplished in the past. In New York State last year there were five members of the Legislature who were physicians and much pioneer work was accomplished by the Public Health Committee of which they were all members.

This revision will be an expensive undertaking, and it is hoped the Health Officers of the State will realize its importance and do their part toward bringing the work to a successful issue. It is not expected to be completed inside of two or three years.

The Sanitary Officers' Association meeting was held the day previous to the usual conference of State Sanitary Officers, which was also well attended; over eight hundred being registered. All declared themselves unusually pleased with the New York meeting, as it affords unusual opportunities for instruction and investigations. It is hoped that Commissioner Porter will see fit in future to hold the conferences in New York at least every alternate year.

---

The Medical Society of the County of Niagara held its annual meeting at Lockport, November 15. With the coöperation of the State Department of Health, a public meeting was held at which Dr. W. D. Alsever of New York spoke of various common ways of spreading infections. The following officers were elected: President, Dr. E. S. N. Ringueberg of Lockport; Vice-President, Dr. C. G. Leo-Wolf of Niagara Falls; Secretary, Dr. L. R. Hurlburt, Cambria; Treasurer, Dr. W. A. Scott, Niagara Falls; Censors: Drs. H. H. Mayne and F. J. Baker of Lockport, and F. Guillemont of Niagara Falls; State Delegate: Dr. C. L. Preisch, Lockport; 8th District Branch Delegate: Dr. E. B. Horton, Niagara Falls.

The I. C. I. Chapter of the Nu Sigma Nu Fraternity held its annual smoker at German-American Hall, Buffalo, November 13, 1911.

The December meetings of the Buffalo Academy of Medicine are scheduled as follows: December 5 (Surgery) Unusual Phases of Acute Perforating Gastric Ulcer, Dr. Ellsworth Elliott, Jr., New York.

December 12 (Medicine) Short History of the Ernest Wende Hospital and Differential Diagnosis of Infectious Diseases, Dr. Walter S. Goodale; Treatment of Infectious Diseases, Dr. Jesse N. Roe.

December 19 (Obstetrics and Gynaecology) Pregnancy and Childbirth as Factors in the Production of Rectal Diseases, Dr. D. C. McKenney; Obstetric Adjuvants, Dr. George R. Stearns.

December 26 (Pathology) Pathology of Pneumonia, Dr. William McCallum, New York.

